# Autophagy receptors as viral targets

**DOI:** 10.1186/s11658-021-00272-x

**Published:** 2021-06-24

**Authors:** Päivi Ylä-Anttila

**Affiliations:** 1 Department of Cell and Molecular Biology, Karolinska Institutet, Stockholm, Sweden; 2 Present Address: Department of Medicine, Solna, Microbial Pathogenesis Unit, Karolinska Institutet, Stockholm, Sweden; 3 Present Address: Division of Neurology, Karolinska University Hospital, Stockholm, Sweden

**Keywords:** Autophagy, Infection, Receptor, Viruses, Cargo, Xenophagy

## Abstract

Activation of autophagy is part of the innate immune response during viral infections. Autophagy involves the sequestration of endogenous or foreign components from the cytosol within double-membraned vesicles and the delivery of their content to the lysosomes for degradation. As part of innate immune responses, this autophagic elimination of foreign components is selective and requires specialized cargo receptors that function as links between a tagged foreign component and the autophagic machinery. Pathogens have evolved ways to evade their autophagic degradation to promote their replication, and recent research has shown autophagic receptors to be an important and perhaps previously overlooked target of viral autophagy inhibition. This is a brief summary of the recent progress in knowledge of virus-host interaction in the context of autophagy receptors.

## Introduction

Autophagy is an evolutionarily conserved degradation and recycle process that in eukaryotes serves as an important part of the innate immune response. The innate immune system is the first cellular response to invading microbes including viruses and entails the recognition of molecules typical for pathogens termed pathogen-associated molecular patterns (PAMPs) by germline-encoded host sensors called pattern recognition receptors (PRRs). Through diverse adaptors, PRRs activate the nuclear factor of kappa light polypeptide gene enhancer in B-cells (NFKB) for inflammatory cytokine production and interferon (IFN) regulatory factors (IRFs) for IFN production [1]. The retinoic acid-inducible gene I (RIG-I) like receptors (RLRs) are PRRs involved in RNA sensing and the cytosolic DNA sensor cyclic GMP-AMP (cGAMP) synthase (cGAS) is the PRR recognizing dsDNA. cGAS produces cGAMP that subsequently activates the endoplasmic reticulum (ER)-associated stimulator of IFN genes protein (STING) [1]. The finding that the cGAS-STING pathway activates autophagy separately from the IFNs and inflammatory cytokines revealed autophagy as a primordial and highly conserved innate immunity pathway pre-dating the emergence of the type I IFN pathway in vertebrates [2]. Autophagy is additionally induced by the protein kinase R (PKR) in response to double stranded RNA through phosphorylation of the elongation and initiation factor α (eIF2 α) [3].

There are three main types of autophagy recognized today, namely macroautophagy, microautophagy and chaperone-mediated autophagy [4, 5]. This review will focus on a subtype of macroautophagy (hereafter autophagy), named selective autophagy, that relies on specialized receptors for specificity [6]. Autophagosomes originate from a cup-shaped membrane structure called the phagophore which then elongates to envelop cargo and finally fuses with lysosomes for degradation. Conserved sets of protein complexes are required for the formation of autophagosomes: the ULK1/2 kinase complex, the Beclin 1 (BECN1)- phosphatidylinositol 3-kinase class III (PIK3C3)/VPS34 kinase complex, the ATG9A membrane cycling system and the two sequentially acting ubiquitin-like conjugation systems ATG12-ATG5-ATG16L1 complex and microtubule associated protein 1 light chain 3 (MAP1LC3/LC3) conjugation to phosphatidylethanolamine [5]. Unlike non-selective autophagy which entails sequestration of intracellular material for example as a response to nutrient deprivation, selective autophagy targets specific cargoes such as damaged organelles or invading pathogens (xenophagy) that are marked for destruction with ubiquitin or galectin tags and are consequently recognized by autophagy receptors including sequestosome 1 (SQSTM1/p62), neighbor of BRCA1 (NBR1), calcium binding and coiled-coil domain-containing protein 2 (CALCOCO2)/nuclear dot 10 protein 52 (NDP52), TRAF6-binding protein (T6BP)/Tax1-binding protein 1 (TAX1BP1) and optineurin (OPTN) [7]. These receptors then bind to autophagosome membrane attached LC3 through their LC3-interacting regions (LIRs) to engage the autophagic machinery. Even though mostly receptors recognize tagged cargoes, interactions independent of post-translational modifications have been reported such as SQSTM1/p62 interaction with the Sindbis virus (SIN) capsid protein [8] and a direct interaction with the Epstein-Barr virus (EBV) deubiquitinase enzyme BamH1 fragment left open reading frame-1 (BPLF1) [9]. Autophagic receptors are key players in the cell defense strategy against invading pathogens since all have been demonstrated to be able to target invaders to lysosomal degradation [10]. It is therefore not surprising that more and more investigations find microbes specifically targeting these key molecules.

### Receptor inactivation by targeting to proteasomal degradation

**Fig. 1:**
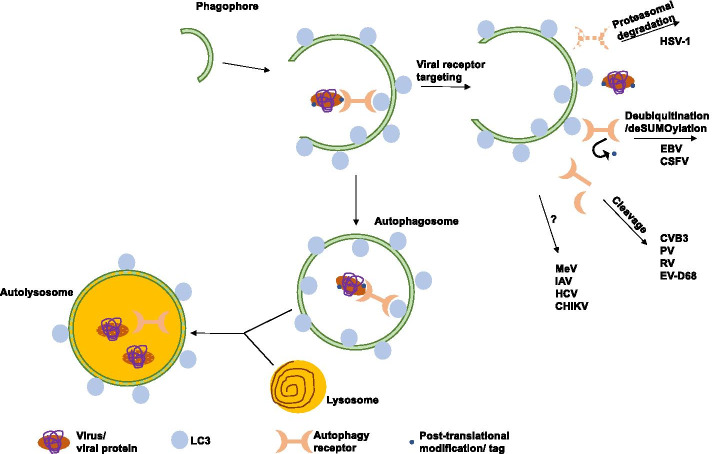
Viral strategies to target autophagy receptors: targeting for proteasomal degradation, herpes simplex virus type 1 (HSV-1); deubiquitination/deSUMOylation, Epstein–Barr virus (EBV), classical swine fever virus (CSFV); proteolytic cleavage, coxsackievirus B3 (CVB3), poliovirus (PV), rhinovirus (RV), enterovirus D68 (EV-D68); binding and/or unknown mechanism, measles virus (MeV), influenza A virus (IAV), hepatitis C virus (HCV) and chikungunya virus (CHIKV)

Recent work by several independent groups has contributed to our knowledge about viral strategies to overcome autophagic degradation. In order to escape the vigorous cellular defense responses viruses need to either remove the tags marking degradation or, more efficiently, functionally inactivate the receptors mediating the physical connection to the autophagic machinery (Fig. 1). Several ways how this inactivation is achieved have been reported (Summary in Table1). During the early phases of the human alpha herpesvirus 1 (HSV-1) infection the autophagic receptors SQSTM1/p62 and OPTN are downregulated by a mechanism that involves their proteasomal degradation rather than activation of the autophagic pathway [11]. The viral protein responsible for this downregulation was identified as infected cell protein 0 (ICP0) and, interestingly, the downregulation did not involve its E3 ubiquitin ligase activity. Indeed, an intriguing possibility is that the ligase could replace an endogenous deubiquitinase which then would lead to a higher ubiquitin status and further to degradation of the receptor. Another possibility which should be explored is the possible involvement of another ligase in the same complex. Even though the molecular details of the functional elimination of these receptors are still unknown, expression of exogenous SQSTM1/p62 in infected cells resulted in decreased virus yields, whereas depletion of either SQSTM1/p62 or OPTN led the cells to mount greater antiviral responses [11], suggesting that SQSTM1/p62 and OPTN might negatively regulate innate immune responses. The downregulation of SQSTM1/p62 and OPTN in HSV-1 infection seems to happen early on during infection without the requirement of viral replication, requiring calcium and ICP0 cytoplasmic localization. The importance of calcium for the functional activity of ICP0 is currently unknown, but it may trigger signaling pathways or activate kinases that are needed for ICP0 activation. The authors speculate that evading the host could be the prerequisite for the receptor down-regulation [11]. Another autophagic receptor linked to HSV-1 infection was reported much earlier and involved the nuclear removal of CALCOCO2/NDP52, but the details and significance of this redistribution of the receptor are not known [12].

**Table 1:** Summary of known viral proteins, targeted autophagic receptor and mechanism of inhibition/activation of autophagy

Virus	Viral protein	Autophagic receptor	Inhibition (I)/ activation (A) of autophagy	Mechanism
HSV-1	ICP0	SQSTM1/p62 OPTN	I	Proteosomal targeting
HSV-1	Unknown	CALCOCO2/NDP52	I	Unknown
EBV	BPLF1	SQSTM1/p62	I	Deubiquitination
CSFV	Unknown	CALCOCO2/NDP52	A	Deubiquitination/deSUMOylation
CVB3	_2C_pro _3C_pro	SQSTM1/p62 NBR1	I	Proteolytic cleavage
PV	Unknown	SQSTM1/p62	I	Proteolytic cleavage
RV1A	Unknown	SQSTM1/p62	I	Proteolytic cleavage
EV-D68	Protease 3C	SQSTM1/p62	I	Proteolytic cleavage
MeV	MeV-N	T6BP/TAX1BP1	A	Unknown
MeV	MeV-C, MeV-V	CALCOCO2/NDP52	A	Unknown
IAV	PB1-F2	CALCOCO2/NDP52	A	Unknown
HCV	NS3	CALCOCO2/NDP52	A	Unknown
CHIKV	nsP2	CALCOCO2/NDP52	A	Unknown

### Receptor inactivation by proteolytic cleavage

The C-terminus of SQSTM1/p62 is known to exert its cargo binding ability through the C-terminal ubiquitin association domain (UBA), whereas the N-terminal Phox/Bem1p (PB1) domain is important in the process of oligomerization. The Coxsackievirus 3B (CVB3) protease 2A^pro^ was reported previously to target SQSTM1/p62 following CVB3 infection, resulting in cleavage of the protein at amino acid 241 and separation of the PB1 domain from the LIR and UBA domains. Rendering the receptor inactive to function in selective autophagy even though its ability to interact with LC3 through the LIR was unaltered [13]. The ability of the cleavage fragments to form protein aggregates was also greatly decreased, as was the ability of the C-terminal fraction to interact with ubiquitinated proteins. In addition to the receptor function, SQSTM1/p62 fragments also lost the ability to activate the NFKB pathway but retained the ability to stabilize antioxidant transcription factor nuclear factor (erythroid-derived 2)-like 2 (NFE2L2) [13]. The same group recently discovered that NBR1 was also cleaved at two sites, E682 and G402, by two viral proteases, 2A^pro^ and 3C^pro^, generating two cleavage products of about 50 and 100 kDa respectively [14]. The NBR1 cleavage was confirmed to be caspase activity independent. Additionally, the C-terminal cleavage products of both SQSTM1/p62 and NBR1 were observed to cause dominant-negative regulatory effects against the function of native proteins in the clearance of ubiquitin conjugates. However, in the case of NBR1 this was observed with only the 3C^pro^-induced C-terminal fragment but not the 2A^pro^-induced fragment [14]. Finally, SQSTM1/p62 and NBR1 were shown to be mutually regulated so that no compensatory effect of one with the other was observed. The authors hypothesize that the dominant-negative effects from the C-terminal cleavage products are due to competition of binding to LC3 and ubiquitin chains. In light of findings on the UBA domain self-association during inactivity in the absence of ubiquitin [15] it should also be considered possible that the dominant-negative effects arise from self-association of endogenous proteins and the expressed C-terminal fragments.

A more recent study on enterovirus D68 (EV-D68) found a similar cleavage based strategy to be common among picornaviruses by testing SQSTM1/p62 cleavage upon infection with EV-D68, poliovirus 1 (PV), rhinovirus 1A (RV1A) and CVB3 as a positive control [16]. The authors concluded that the significance of the finding is twofold; not only does the autophagic cargo escape degradation (inhibition of degradation by selective autophagy), but the cleavage of SQSTM1/p62 reduces the amount of full length protein and renders the interpretation of the SQSTM1/p62 band in immunoblots as an autophagy marker unreliable for picornavirus infections [16].

Even though mostly autophagic receptors have been thought to mediate an antiviral role, CALCOCO2/NDP52 was recently reported to act in a pro-viral manner promoting viral replication through inhibition of the type I IFN signaling by autophagy-mediated clearance of the mitochondrial antiviral signaling (MAVS) [17]. CALCOCO2/NDP52, like SQSTM1/p62 and NBR1, is cleaved after CVB3 infection by the viral proteinase 3C at Q139, separating the N-terminal skeletal and kidney-enriched inositol phosphatase (SKIP) carboxyl homology (SKICH) and LC3C-interacting region (CLIR) from its C-terminal LIR, coiled-coil (CC), and ubiquitin-binding zinc finger (UBZ) domains. Whereas the N-terminal fragment was unstable and was eliminated through proteasomal degradation, the stable C-terminal fragment retained the pro-viral mechanism of the full length CALCOCO2/NDP52 by MAVS down-regulation mediated type I IFN inhibition. Interestingly, the C-terminal fragment also retained its ability to bind exogenously expressed LC3 and ubiquitin, indicating the ability to act as an autophagy receptor [17]. However, functional investigations are needed to confirm the function in autophagy and as a xenophagic receptor.

### Receptor inactivation by ubiquitin/SUMO modulation

Modification of the cellular ubiquitin and ubiquitin-like machineries is a well-known viral strategy to modulate several intracellular pathways. We recently identified an EBV deubiquitinase as a selective autophagy inhibitor targeting the autophagic receptor SQSTM1/p62 [9]. SQSTM1/p62 is the most thoroughly studied of the autophagic receptors and much is known about its structure and function as an important link between the ubiquitin–proteasome system and autophagy [18]. SQSTM1/p62 function as an autophagic receptor is heavily dependent on the covalently attached ubiquitin on its amino acid residues with special importance of the PB1 domain K7 ubiquitination, which blocks self-interaction with the D69 residue and hence oligomerization [19, 20], and K420 ubiquitination, which prevents interaction with the E409 residue promoting the UBA open conformation to allow cargo binding [21]. Similarly, mutations of these residues cause dominant negative and positive SQSTM1/p62 proteins. Whereas mutation of K7 or D69 disrupts the oligomerization property causing an autophagy deficient mutant [20], mutations in K420 or E409 render the UBA open in the lack of self-association [15, 21, 22]. The N-terminal domain of the large tegument protein BPLF1 encodes a conserved cysteine protease with ubiquitin- and neuronal precursor cell-expressed developmentally down-regulated protein 8 (NEDD8)-specific deconjugase activity [23]. BPLF1 is expressed during the productive virus cycle and, as part of the viral tegument, is delivered to the host cytoplasm upon primary infection [24]. We observed that BPLF1 bound to and deubiquitinated SQSTM1/p62, whereas the catalytic mutant BPLF1 C61A caused strong ubiquitination of precipitated endogenous SQSTM1/p62 [9]. The strong ubiquitination in the presence of the catalytic mutant was due to K48-linked ubiquitin chains added to SQSTM1/p62. However, the precise residues affected and the possible interaction with a ubiquitin ligase remain unresolved. The deubiquitination of the autophagic receptor correlated with failure of LC3 recruitment to SQSTM1/p62 positive structures and with accumulation of a known model of selective autophagic cargo, mutant huntingtin with polyglutamine repeats (HTTPQ) formed aggregates [9]. The accumulation of HTTPQ aggregates was overcome by overexpression of either the dominant active mutant of SQSTM1/p62, the E409A, K420R mutant which does not need K420 ubiquitination for UBA activation and cargo binding, or wild type SQSTM1/p62. Interestingly, the autophagy deficient SQSTM1/p62 mutant, oligomerization deficient SQSTM1/p62 K7A, was unable to rescue the aggregation phenotype [9].

The classical swine fever virus (CSFV) was recently reported to cause down-regulation of CALCOCO2/NDP52 as well as decreased ubiquitination and SUMOylation (small ubiquitin-like modifier) of the receptor or its binding partners in a Parkin dependent manner [25]. Parkin was upregulated and Parkin, or Parkin associated proteins, were ubiquitinated as a response to CSFV infection. CALCOCO2/NDP52 silencing decreased CSFV replication measured by viral titers, RNA copy numbers and reduction of viral protein N^pro^, indicating a positive role for CALCOCO2/NDP52 in CSFV replication [25]. CSFV structural protein E2 was observed colocalizing with CALCOCO2/NDP52 in PK-15 cells. CALCOCO2/NDP52 silencing also decreased the autophagic markers CD63, LC3 and BECN1; however, colocalization between LC3 and ubiquitin was also decreased. CALCOCO2/NDP52 silencing during CSFV infection revealed that CALCOCO2/NDP52 promotes the colocalization between CSFV E2 protein and CD63 as well as ubiquitin [25]. The viral protein responsible for the CALCOCO2/NDP52 ubiquitin modulation is yet to be discovered.

### Receptor interaction by yet unknown mechanisms

T6BP/TAX1BP1 and CALCOCO2/NDP52 were recently identified as essential components required for autophagy maturation mediated measles virus (MeV) replication [26]. MeV is known to exploit the autophagic pathway and to induce a complete autophagic flux to improve its replication [27]. Silencing of T6BP/TAX1BP1 and CALCOCO2/NDP52 with small interfering RNA strongly reduced the ability of MeV to produce infectious particles in infected cells while viral entry remained unaltered. Interestingly, OPTN and SQSTM1/p62 silencing did not prevent MeV replication and further, SQSTM1/p62 silencing instead facilitated the replication, indicating a possible protective role for SQSTM1/p62 against MeV infection. T6BP/TAX1BP1 and CALCOCO2/NDP52 were observed to interact with MeV proteins, T6BP/TAX1BP1 with MeV-N and CALCOCO2/NDP52 with MeV-C and MeV-V; however, the molecular mechanism behind the interaction and the consecutive autophagic maturation is yet to be elucidated. This is another example where successful autophagic maturation is not blocked by the virus but rather needed for virus replication. How MeV is able to promote full autophagic flux and at the same time escape autophagic degradation remains an open question. The authors speculate that T6BP/TAX1BP1 and CALCOCO2/NDP52 could target substrates to autophagosomes whose degradation is required for MeV replication to occur, such as infection-induced apoptotic factors [26].

In another study, the yeast two-hybrid (Y2H) approach identified CALCOCO2/NDP52 as a host interactor of the influenza A virus (IAV) protein PB1-F2 [28], which plays an important role in IAV virulence. CALCOCO2/NDP52-PB1-F2 interaction resulted in enhanced NFKB activity mediated by MAVS, and interference with the TANK-binding kinase 1 (TBK1) signaling pathway. The impact on proinflammatory activity was further confirmed by silencing the endogenous CALCOCO2/NDP52 during IAV infection, which resulted in a modest but significant reduction in IFNβ and more pronounced decrease in IL8, TNFα and RIG-I. However, the authors were unable to observe any differences in the autophagic activity of wild type or PB1-F2 deficient mutant virus ∆F2 IAV infected cells [28]. CALCOCO2/NDP52 receptor has additionally been reported to bind hepatitis C virus (HCV) non-structural protein 3 (NS3) and chikungunya virus (CHIKV) non-structural protein 2 (nsP2). The significance of NS3-CALCOCO2/NDP52 binding has not been explored [29, 29]; however, CHIKV seems to induce autophagy for efficient replication through the nsP2-CALCOCO2/NDP52 interaction in human cells but not in mouse embryonic fibroblasts [31].

## Discussion

Although autophagy is known to play both anti-viral and pro-viral roles, it seems to be a common denominator to various viral infections that autophagy is induced at a certain point of the infection but autophagic degradation of progeny virions is inhibited [32, 33]. Sometimes full autophagic flux is needed for maturation or self-regulation of viral factors, or for selective degradation of host proteins [26]. Being a general stress response, autophagy is a common by-product of any infection. Autophagy contributes to the innate immunity responses early in infection to clear invading pathogens [34] and later on as infection proceeds autophagy plays an important role in the establishment of adaptive immunity by facilitating antigen processing for presentation [35]. As major membrane re-organization machinery, autophagy provides physical scaffolds for viral replication and a membrane-bound protective environment for generating progeny with readily available metabolites for repurposing. After assembly, autophagy can be subverted to facilitate non-lytic viral exit and spread [36]. Targeting of autophagic receptors seems to serve dual roles depending on infection and virus. It is noteworthy that the better understood examples summarized here describe viral targeting of SQSTM1/p62, NBR1 and OPTN, resulting in inhibition of selective autophagy [11, 14, 17], whereas targeting of T6BP/TAX1BP1 and CALCOCO2/NDP52 by different viruses seems to result in promotion of full autophagic flux [26, 28–30]. T6BP/TAX1BP1 and CALCOCO2/NDP52 have been reported to have dual roles in autophagy, both serving as cargo receptors and functioning in autophagosome maturation [37], which could be connected to the different outcome compared to SQSTM1/p62, NBR1 and OPTN targeting.

Since autophagic receptors exhibit pleiotropic functions there are several possibilities, besides direct prevention of autophagic degradation, for explanations as to why viruses might want to either inhibit or activate them. SQSTM1/p62 and OPTN are both involved in clearance of mitochondria via mitophagy, but the role of mitophagy in HSV-1 infection is currently unknown. Mitochondria have a central role in mediating innate immune responses via the mitochondrial antiviral-signaling protein (MAVS) [38], and aging-related mitochondrial dysfunction is a hallmark of inflammaging. One of the driving forces of inflammaging is the chronic burden on immune cells caused by latent viral infections such as the human cytomegalovirus (HCMV) [39]. The balance between mitochondrial biogenesis and mitophagy is vital for mitochondrial homeostasis and regulated through expression of the master regulator of mitochondrial biogenesis, peroxisome proliferator-activated receptor gamma coactivator 1-alpha (PGC-1α), which induces ULK1 expression [40, 41]. It is important to investigate the consequences of mitochondrial quality control in autophagy function during viral infections.

SQSTM1/p62 and T6BP/TAX1BP1 both regulate NFKB signaling, which is important in HSV-1 replication [42] and could explain NFKB upregulation by the virus through SQSTM1/p62 elimination. SQSTM1/p62 also has a role in inflammasome regulation [43] as well as induction of antioxidant responses through the Keap1-Nrf2 pathway [44], which might be counteracted by HSV-1 through targeting of the receptor. As an addition to the role in mitophagy, OPTN has been implicated in several protein and membrane trafficking processes as well as signaling events critical to the innate immune response such as NFKB activity regulation and interferon production [45].

NBR1 has similar domain architecture as SQSTM1/p62 and the proteins act co-operatively in selective autophagy. Interestingly, NBR1 is larger than SQSTM1, has additional domains and is more ubiquitous among species than SQSTM/p62, but much less is still known about NBR1 function. In mice NBR1 is an important mediator of T-cell maturation [46]. T6BP/TAX1BP1 and CALCOCO2/NDP52, like OPTN, are myosin VI binding proteins involved in membrane trafficking, but especially the role of T6BP/TAX1BP1 is still largely unknown. In addition to binding myosin VI, T6BP/TAX1BP1 and CALCOCO2/NDP52 also bind each other and have been suggested to modulate cytokine signaling and membrane transport with actin filament organization and cell adhesion [47]. There are several recent studies on CALCOCO2/NDP52 that have revealed important molecular insights on its role as the recruiter of the early autophagic protein complexes to the cargo, initiating selective autophagy [48–50], and a recent review summarizes its role in microbial infections, including viruses [51]. CALCOCO2/NDP52 has a role in innate immunity through NFKB and type I IFN regulation, which can be another explanation for CSFV CALCOCO2/NDP52 targeting.

## Conclusion

The current understanding of the autophagy pathway as a viral target encompasses the process in its entirety. Autophagy is not only inhibited but is regulated by viruses at several points from signaling all the way through membrane elongation and closure to maturation and recycling of autolysosome content and membrane, and has been thoroughly discussed in recent reviews [1, 52]. It is clear that autophagy receptor targeting is an important viral strategy to modulate the host signaling pathways, and viruses employ several approaches, such as proteasomal targeting, proteolytic cleavage and modulation of post-translational modifications, to functionally inactivate these receptors. Dissecting the molecular details of virus interaction with the host intracellular pathways remains exceedingly important for drug repurposing and development. Preclinical investigations around the current pandemic of coronavirus disease 2019 (COVID-19) caused by severe acute respiratory syndrome coronavirus 2 (SARS-CoV-2) highlights autophagy as an important drug target, as a large proportion of these drugs act on the recycling pathway [53]. Future investigations will aid in understanding the development of viral diseases and in mapping targets for specific antiviral therapies.

## Data Availability

Not applicable.

## Abbreviations

PAMP: Pathogen-associated molecular pattern
PRR: Pattern recognition receptor
NFKB: Nuclear factor of kappa light polypeptide gene enhancer in B-cells
IFN: Interferon
RIG-I: Retinoic acid-inducible gene I
RLR: RIG-I like receptor
cGAMP: Cytosolic DNA sensor cyclic GMP-AMP
cGAS: CGAMP synthase
STING: Stimulator of IFN genes protein
PKR: Protein kinase R
eIF2α: Elongation and initiation factor 2α
BECN1: Beclin1
PIK3C3: Phosphatidylinositol 3 kinase class III
ATG: Autophagy related gene
MAP1LC3: Microtubule associated protein 1 light chain 3
SQSTM1: Sequestosome 1
NBR1: Neighbor of BRCA 1
CALCOCO2: Calcium binding and coiled-coil domain-containing protein 2
NDP52: Nuclear dot 10 protein 52
T6BP: TRAF6-binding protein
TAX1BP1: Tax1-binding protein 1
OPTN: Optineurin
LIR: LC3-interacting region
HSV-1: Herpes simplex virus 1
ICP0: Infected cell protein 0
UBA: Ubiquitin associated domain
PB1: Phox/Bem1p
CVB3: Coxsackievirus 3
EV-D68: Enterovirus D68
PV: Poliovirus
RV1A: Rhinovirus 1A
MAVS: Mitochondrial antiviral signaling
SKIP: Skeletal and kidney-enriched inositol phosphatase
SKICH: SKIP carboxyl homology
CLIR: LC3C-interacting region
CC: Coiled coil
UBZ: Ubiquitin-binding zinc finger
EBV: Epstein–Barr virus
BPLF1: BamH1 fragment left open reading frame-1
NEDD8: Neuronal precursor cell-expressed developmentally down-regulated protein 8
HTT: Huntingtin
PQ: Polyglutamine
CSFV: Classical swine fever virus
SUMO: Small ubiquitin-like modifier
MeV: Measles virus
IAV: Influenza A virus
Y2H: Yeast two hybrid
TBK1: TANK binding kinase 1
CHIKV: Chikungunya virus
NS3: Non-structural protein 3
nsP2: Non-structural protein 2
HCMV: Human cytomegalovirus
PGC-1α: Peroxisome proliferator-activated receptor gamma coactivator 1-alpha
SARS-CoV-2: Severe acute respiratory syndrome coronavirus 2
